# Unusual Complexes of P(CH)_3_ with FH, ClH, and ClF

**DOI:** 10.3390/molecules25122846

**Published:** 2020-06-19

**Authors:** Janet E. Del Bene, Ibon Alkorta, José Elguero

**Affiliations:** 1 Department of Chemistry, Youngstown State University, Youngstown, OH 44555, USA; 2 Instituto de Química Médica (IQM-CSIC), Juan de la Cierva, 3, E-28006 Madrid, Spain; iqmbe17@iqm.csic.es

**Keywords:** hydrogen bonds, halogen bonds, dipole interactions, coupling constants

## Abstract

Ab initio MP2/aug’-cc-pVTZ calculations have been performed to determine the structures and binding energies of complexes formed by phosphatetrahedrane, P(CH)_3_, and HF, HCl, and ClF. Four types of complexes exist on the potential energy surfaces. Isomers **A** form at the P atom near the end of a P-C bond, **B** at a C-C bond, **C** at the centroid of the C-C-C ring along the C_3_ symmetry axis, and **D** at the P atom along the C_3_ symmetry axis. Complexes **A** and **B** are stabilized by hydrogen bonds when FH and ClH are the acids, and by halogen bonds when ClF is the acid. In isomers **C**, the dipole moments of the two monomers are favorably aligned but in **D** the alignment is unfavorable. For each of the monomers, the binding energies of the complexes decrease in the order **A** > **B** > **C** > **D**. The most stabilizing Symmetry Adapted Perturbation Theory (SAPT) binding energy component for the **A** and **B** isomers is the electrostatic interaction, while the dispersion interaction is the most stabilizing term for **C** and **D**. The barriers to converting one isomer to another are significantly higher for the **A** isomers compared to **B**. Equation of motion coupled cluster singles and doubles (EOM-CCSD) intermolecular coupling constants J(X-C) are small for both **B** and **C** isomers. J(X-P) values are larger and positive in the **A** isomers, negative in the **B** isomers, and have their largest positive values in the **D** isomers. Intramolecular coupling constants ^1^J(P-C) experience little change upon complex formation, except in the halogen-bonded complex FCl:P(CH_3_) **A**.

## 1. Introduction

**Figure 1:**
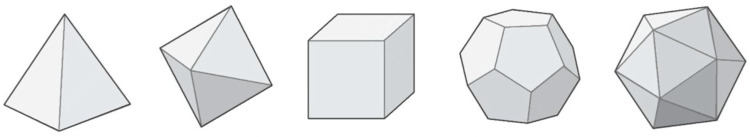
Five *Platonic* structures: tetrahedron, octahedron, hexahedron (cube), dodecahedron, and icosahedron.

Symmetry in Chemistry is one of the cornerstones of chemistry, both in terms of the beauty it bestows on molecules, and its importance for life. There is no question that highly symmetrical molecules are intrinsically beautiful and fascinating. Moreover, symmetry in chemistry, particularly chirality, is important for life itself, as first demonstrated by Pasteur [1,2,3,4]. In addition to the highly symmetrical *Archimedean* structures mainly known thanks to the fullerenes, the five *Platonic* structures which do not have atoms in the center, but only on the periphery, are also intrinsically beautiful. The Platonic structures are illustrated in Figure 1.

Octahedron-type molecules are unknown, [1] but icosahedron geometries are found in boron derivatives such as the dodecaborate dianion [2] and the *closo*-carboranes [3]. The three other structures could exist as pure carbon derivatives, although in some cases, with external substituents needed for stabilization. These include tetrahedrane (CH)_4_, [4,5] cubane (CH)_8_, [6,7,8] and dodecahedrane (CH)_20_ [9,10,11]. Chemists have searched the Periodic Table for structures which could be obtained by replacing C or CR by X or XR. Theoretical studies of tetrahedrane molecules in which C is replaced by elements from Si to Pb and from B to Tl, [12,13] CR is replaced by SiR or GeR, [9] or CH is replaced by N, have demonstrated that these are stable systems [14,15,16].

An early study of replacing CR by P was carried out on phosphacubanes by Reitz et al. [17]. Di-tert-butyldiphosphatetrahedrane has been recently synthesized and characterized by Wolf et al. [18]. White phosphorus, P_4_, which has a tetrahedral structure, has been the subject of many theoretical studies [19,20,21,22]. As is the case for C_4_, all tetrahedral (CR)_4_ molecules lack a dipole moment. This usually leads to weaker noncovalent interactions, even in cases in which the interacting molecule may have a large positive σ-hole. Yáñez demonstrated that phosphatetrahedrane and diphosphatetrahedrane behave as carbon bases in the gas phase [23]. Boldyrev et al. studied the molecules C*_n_*H*_n_*P_4–*n*_ (*n* = 0–4) and their transformation into planar structures [24]. We have investigated the P_4_ molecule in complexes stabilized by hydrogen, halogen, and pnicogen bonds [25]. The “elusive phosphatetrahedrane” was finally isolated by Cummins et al. [26] in the form of the tris-*tert-*butyl derivative.

In the present study, we have searched the potential energy surfaces of phosphatetrahedrane, P(CH)_3_ interacting with FH, ClH, and ClF. The structures and binding energies of the isomers that exist on these surfaces have been determined, as well as the transition structures that convert one equilibrium isomer to another. To gain further insight into the binding energies of these complexes, SAPT analyses have been carried out. Finally, EOM-CCSD spin–spin coupling constants across intermolecular bonds have been computed for all complexes. It is the purpose of this paper to present the results of this study.

## 2. Results and Discussion

### 2.1. Parent Molecule P(CH)_3_ and Its Molecular Electrostatic Potential (MEP)

The parent molecule P(CH)_3_ is pyramidal with C_3v_ symmetry. Although the experimental structure of this molecule is not known, a derivative P(C-t-butyl)_3_ has been synthesized. An X-ray diffraction study of this molecule reported P-C distances of 1.837 to 1.860 Å, and C-C bond distances of 1.467 to 1.481 Å. The computed P-C distances of P(CH)_3_ are 1.857 Å and the C-C distances are 1.465 Å. In addition, the one-bond coupling constant ^1^J(P-C) has been measured experimentally and found to have an unsigned value of 37.9 Hz. The computed EOM-CCSD value of ^1^J(P-C) for P(CH)_3_ is –40.3 Hz. Thus, the computed structure and P-C coupling constant for P(CH)_3_ are consistent with the experimental values for the t-butyl derivative.

**Figure 2:**
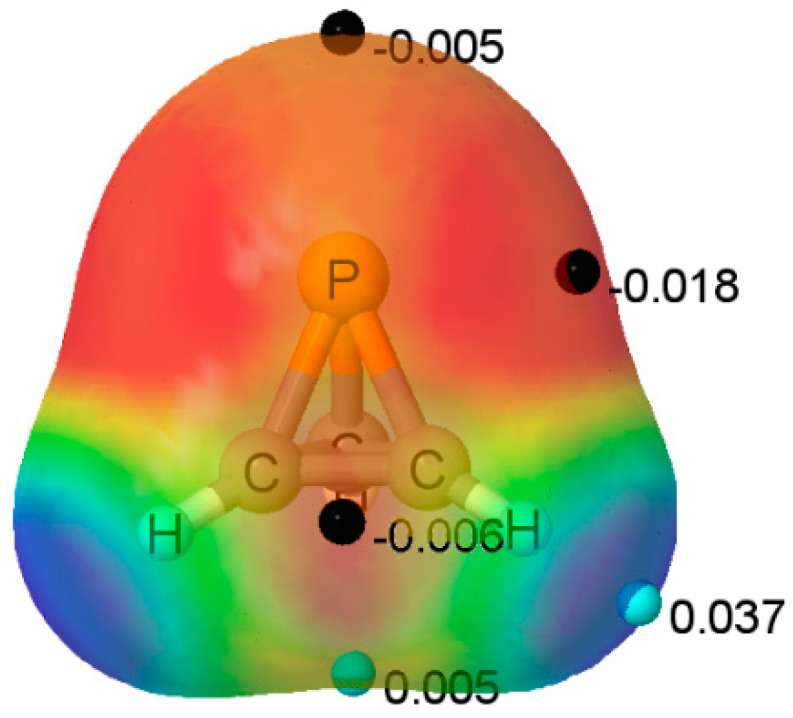
Molecular Electrostatic Potential (MEP) of P(CH)_3_ on the 0.001 au electron density isosurface. The color coding ranges from red < −0.01 au to blue > +0.04 au. The locations and values of local minima and maxima of interest on this surface are indicated by black and light blue dots, respectively.

**Figure 3:**
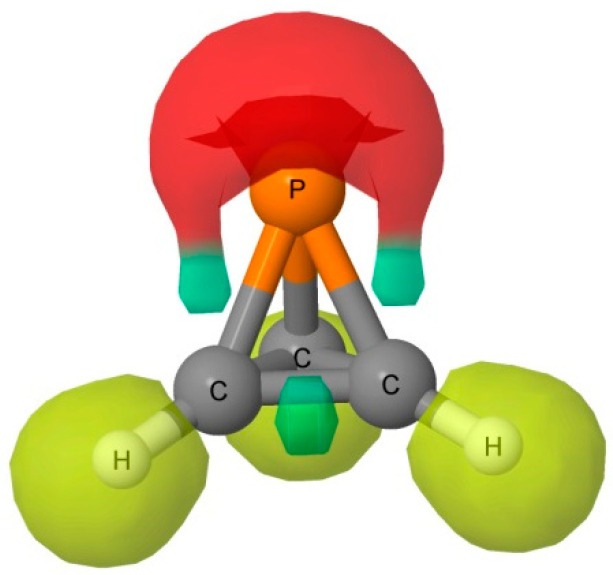
ELF 0.8 isosurface. Yellow, red and green indicate the hydrogenoid, monosynaptic, and disynaptic basins, respectively.

The MEP of the P(CH)_3_ molecule is illustrated in Figure 2. A triply degenerate MEP minimum with a value of −0.018 au is located at the P atom almost perpendicular to the C_3_ symmetry axis. Three additional triply degenerate minima with values of −0.006 au are associated with the midpoint of each C-C bond in the ring. There is also a minimum of −0.005 au located on the C_3_ axis at the P atom. At these minima, P(CH)_3_ should act as an electron donor. There are also two unique maxima on this surface. One is a three-fold degenerate MEP maximum associated with the three C-H bonds. The second maximum has a value of 0.005 au, and is found below the centroid of the C-C-C ring on the C_3_ symmetry axis. Bond formation in this region should have P(CH)_3_ acting as the acid, that is, as an electron acceptor. The Electron Localization Function (ELF) analysis indicates that the deepest MEP minima on the 0.001 au electron density isosurface of −0.018 au arises from simultaneous contributions from the P-C bond basins and the P lone pair, as illustrated in Figure 3.

### 2.2. Overview of the Equilibrium Binary Complexes

**Figure 4:**
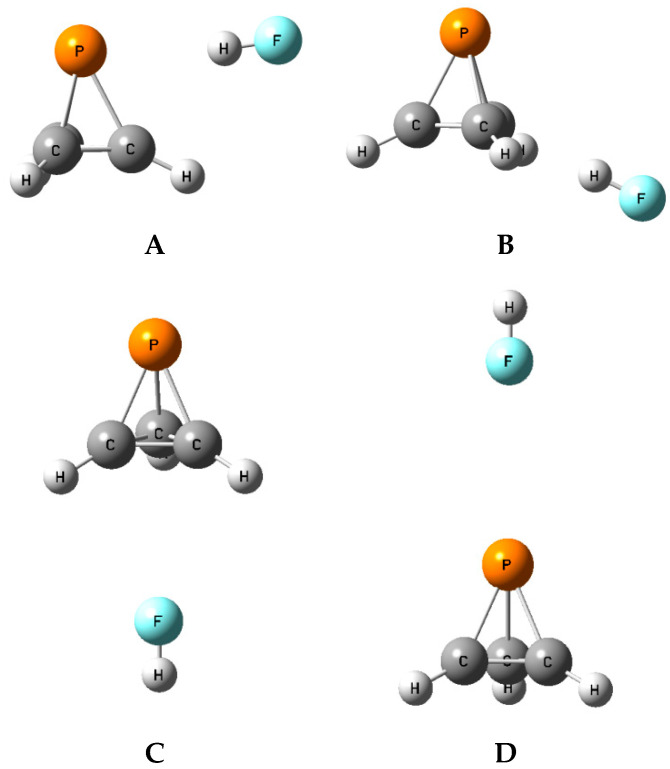
The equilibrium isomers (**A**–**D**) of FH:P(CH)_3_.

On the XY:P(CH)_3_ surfaces with each of the acids, four unique equilibrium complexes have been found. XY stands for FH, ClH, and FCl in these complexes. Different starting structures based on the electrostatic potential of the phosphatetrahedrane and its symmetry have been used in the search for equilibrium structures. The structures, total energies, and molecular graphs of the equilibrium complexes are reported in Tables S1–S4 of the Supplementary Materials, and the structures of equilibrium complexes **A**, **B**, **C**, and **D** are illustrated for FH:P(CH)_3_ in Figure 4. In what follows, these structures will be discussed in the order **A**, **B**, **C**, **D**. In addition, two other structures **A**-tr and **B**-tr are stationary points on the potential surfaces, and will also be discussed. We were not able to find any complexes on the potential energy surfaces in which P(CH)_3_ formed C-H—X hydrogen bonds even when the starting geometries included the hydrogen-bonding interaction.

### 2.3. Binary Complexes A

The binary complexes **A** form at the local minimum identified in the MEP with a value of −0.018 au. The acid lies in the plane defined by the adjacent P-C and C-H bonds, so there are three such equilibrium structures on the potential energy surface. The structures, total energies, and molecular graphs of these complexes are reported in Table S1. The binary complexes of P(CH)_3_ with FH and ClH are stabilized by F-H⋯P and Cl-H⋯P hydrogen bonds, while the complex with ClF is stabilized by a P⋯Cl halogen bond.

Table 1 presents the binding energies, intermolecular distances, selected angles, charge-transfer energies, and coupling constants across intermolecular bonds for these complexes. The most stable complex is the halogen-bonded complex FCl:P(CH)_3_ with a binding energy of 32 kJ mol^−1^, followed by the hydrogen-bonded complexes FH:P(CH)_3_ and ClH:P(CH)_3_ with binding energies of 20 and 17 kJ mol^−1^, respectively. The corresponding intermolecular F-P and Cl-P distances are 3.41 and 3.87 Å in the hydrogen-bonded complexes, respectively, and 2.55 Å in the halogen-bonded complex. The C-P-X angles vary between 65 and 77°, consistent with the observation made from the MEP that complex formation should occur at P in a direction almost perpendicular to the local C_3_ axis. The H-X-P angles of 5° indicate that the hydrogen bonds deviate only slightly from linearity, while the P-Cl-F angle of 169° aligns the Cl-F molecule to accept a pair of electrons from P through the σ-hole on Cl for the formation of the halogen bond. In the halogen-bonded complex, there is a significant elongation of the P-C bond that interacts with Cl, as evident from Table 1. While this bond length is 1.881 and 1.875 Å in the complexes with FH and ClH, respectively, it increases dramatically to 1.946 Å in the complex with FCl. Further discussion of bond length changes and ^1^J(P-C) coupling constants for isomers **A**, **B**, **C**, and **D** will be given below.

Charge-transfer energies P_lp_―σ*X-H of 17.6 and 16.4 kJ mol^−1^ are found for the hydrogen-bonded complexes. This energy changes dramatically in the halogen-bonded FCl:P(CH)_3_ complex. The largest charge-transfer energy arises from electron donation by a σ(P-C) bonding orbital to an antibonding Cl-F orbital, with an energy of 83 kJ mol^−1^. There is a second P_lp_―σ*Cl-F charge-transfer with an energy of 36.5 kJ mol^−1^. The smallest charge-transfer energy of 17 kJ mol^−1^ is a reverse charge-transfer, from the chlorine lone pair to an antibonding P-C orbital.

Spin–spin coupling constants ^2h^J(X-P) are also reported in Table 1, and have values of 28 and 3 Hz for the hydrogen-bonded complexes with FH and ClH, respectively. ^1x^(Cl-P) is 232 Hz for the halogen-bonded FCl:P(CH)_3_ complex. The components of these coupling constants are reported in Table S5 and indicate that although these coupling constants are dominated by the Fermi contact (FC) term, the paramagnetic spin orbit (PSO) and spin dipole (SD) terms make non-negligible contributions. Figure S1 of the Supplementary Materials provides a plot of ^2h^J(F-P) versus the F-P distance along the intrinsic reaction path connecting F and P. ^2h^J(F-P) varies from 9 Hz at an F-P distance of 3.71 Å to 64 Hz at a distance of 3.21 Å. The second-order trendline in Figure S1 has a correlation coefficient of 0.998, and suggests that the F-H**^…^**P hydrogen bond is a traditional hydrogen bond. Figure S2 is a plot along the intrinsic Cl-P reaction path for FCl:P(CH)_3_. The third-order trendline has a correlation coefficient of 0.999. The curvature of this trendline at short distances suggests that complexes with these distances would have chlorine-shared halogen bonds [27,28]. Thus, the Cl**^…^**P halogen bond in FCl:P(CH)_3_ isomer **A** would appear to have increased chlorine-shared character.

The properties of the complexes with P(CH)_3_ may be compared to the properties of the same acids with PH_3_. The X-P distances in the complexes XY:P(CH)_3_ are longer than the corresponding distances in the XY:PH_3_ complexes, which are 3.270, 3.800, and 2.183 Å for the complexes with FH, ClH, and ClF, respectively. The binding energies of the complexes FH:PH_3_ and ClH:PH_3_ are 21.4 and 14.9 kJ mol^−1^, respectively, which are similar to those for the corresponding complexes with P(CH)_3_. However, the FCl:P(CH)_3_ binding energy of 31.6 kJ mol^−1^ is significantly less than that of FCl:PH_3_ which is 51.2 kJ mol^−1^. This large difference may be attributed primarily to the weakening of the interacting P-C bond of P(CH)_3_, as indicated by the significant lengthening of this bond.

### 2.4. Binary Complexes B

**Figure 5:**
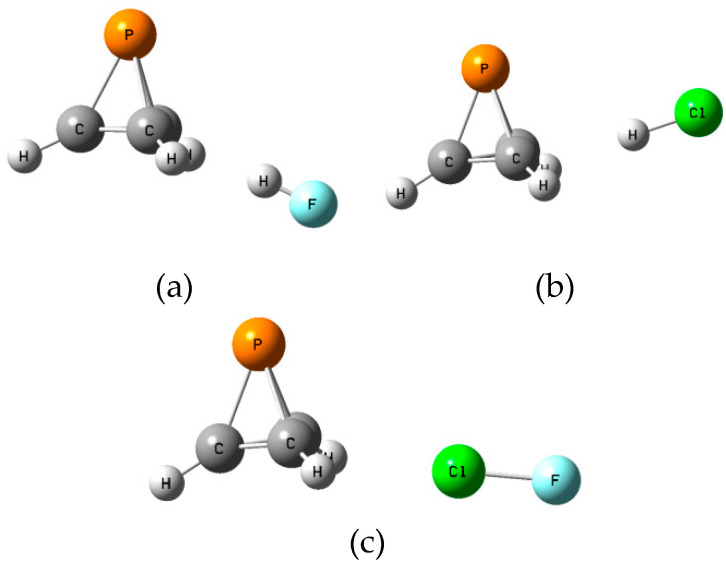
Structures of (**a**) FH:P(CH)_3_, (**b**) ClH:P(CH)_3_, and (**c**) FCl:P(CH)_3_ isomers **B**.

The **B** isomers of the binary complexes XY:P(CH)_3_ arise as a carbon-carbon bond of P(CH)_3_ donates a pair of electrons to XY through its σ-hole. There are three such equilibrium complexes on the XY:P(CH)_3_ surface, one at each C-C bond. Table S2 of the Supplementary Materials provides the structures, total energies, and molecular graphs of these equilibrium complexes. Table 2 reports their binding energies, the X-C distances, and the distances from X to the midpoint of the C-C bond (γ), the P-γ-X angles, charge-transfer energies, and coupling constants across the intermolecular bonds. Figure 5 illustrates the structures of the **B** isomers.

The binding energies of the **B** isomers are less than those of the **A** isomers, and extend over a smaller range, from 14.1 kJ mol^−1^ at a distance of 2.19 Å from Cl to the midpoint of the C-C bond when ClH is the acid, to 17.3 kJ mol^−1^ at a distance of 2.83 Å from the midpoint of the C-C bond to the Cl atom when ClF is the acid. The angles defined by P, the midpoint of the C-C bond, and X are similar when FH and FCl are the acids, but smaller when ClH is the acid, as evident from Figure 5 and Table 2. The distances from X to one of the two C atoms range from 2.92 Å in the complex with ClF to 3.54 Å in the complex with ClH. The charge-transfer energies of these complexes are approximately 2 kJ mol^−1^, and are, therefore, much less than the charge-transfer energies of the **A** isomers. They arise from electron donation by the C-C bond to a σ antibonding X-Y orbital. The order of decreasing charge-transfer energies is the same as the order of decreasing binding energies.

The DFT-SAPT data for the **A** and **B** complexes in Table 3 allow for further insight into the binding energies of these isomers. There is only one destabilizing interaction, and that is the first-order exchange energy which varies from 27 kJ mol^−1^ for ClH:P(CH)_3_ isomer **B** to 270 kJ mol^−1^ for FCl:P(CH)_3_ isomer **A**. The remaining SAPT terms are stabilizing and are sufficient to overcome the exchange term, leading to SAPT binding energies [−ΔE(SAPT)] which range from 10 kJ mol^−1^ for the **B** isomers of ClH:P(CH)_3_ and FCl:P(CH)_3_ to 35 kJ mol^−1^ for the **A** isomer of FCl:P(CH)_3_. The electrostatic interaction term is the most stabilizing term for all of these isomers except ClH:P(CH)_3_ isomer **B**, in which case the dispersion term is the leading stabilizing term. For FCl:P(CH)_3_ isomer **B**, the dispersion term is about 1 kJ mol^−1^ less stabilizing than the electrostatic term. The SAPT binding energies are less than the MP2 binding energies except for FCl:P(CH)_3_ isomer **A** which has a SAPT binding energy that is 3 kJ mol^−1^ greater than the MP2 energy.

Spin–spin coupling constants across intermolecular bonds for the **B** isomers are also reported in Table 2. The coupling constants are negligible at 0.1 and 0.2 Hz for the complexes with HCl and ClF, respectively. The coupling constant with the largest absolute value is ^2h^J(F-C) for FH:P(CH)_3_ with a value of only −1.8 Hz. It is interesting to note from Table S5 that the dominant term for ^2h^J(F-Cl) is the PSO term followed by the SD term. That the FC term is negligible is quite unusual for a hydrogen-bonded complex, but the hydrogen bonds in these **B** complexes are not typical hydrogen bonds.

### 2.5. Binary Complexes C and D

There are two additional equilibrium **C** and **D** isomers on the XY:P(CH)_3_ surfaces. In **C** the dipole moment vectors of XY and P(CH)_3_ are favorably aligned head-to-tail, while in **D** there is an unfavorable alignment, as illustrated in Figure S3 of the Supplementary Materials. One might question why the reverse alignments of FH, ClH, and ClF in **D** which would lead to favorable dipole alignments are not the equilibrium structures. This and related questions will be addressed below.

**Figure 6:**
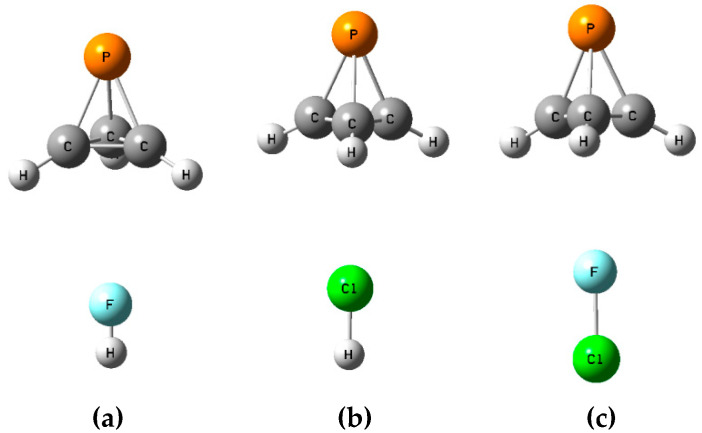
Equilibrium structures **C** for (**a**) FH:P(CH)_3_, (**b**) ClH:P(CH)_3_, and (**c**) FCl:P(CH)_3_.

The structures, total energies, and molecular graphs of the **C** isomers are reported in Table S3 of the Supplementary Materials, and Figure 6 illustrates these isomers. It is noteworthy that it is the F atom and not the Cl that interacts with P(CH)_3_ in the FCl:P(CH)_3_ complex. The structure in which Cl interacts with P(CH)_3_ is not an equilibrium structure. Table 4 provides the binding energies, intermolecular distances, and spin–spin coupling constants of the **C** isomers. It is not surprising that the binding energies no longer follow the order of binding energies found for the hydrogen-bonded **A** and **B** isomers, but decrease instead in the order ClH > ClF > HF. The binding energies range from 4.5 kJ mol^−1^ for FH:P(CH)_3_ to 9.2 kJ mol^−1^ for ClH:P(CH)_3_. The X-C and X-P distances decrease in the order ClF > ClH > FH. That the mostly weakly bound complex with FH has the shortest X-C and X-P distances may be attributed at least in part to the smaller van der Waals radius of F.

Table 4 also reports coupling constants J(X-C) and J(X-P). The coupling constants J(X-C) are small, ranging from 0.0 Hz in the complex with ClF to 3.2 Hz in the complex with FH. Coupling with P is most interesting, since the interaction is through the tetrahedron of P(CH)_3_ at very long distances. J(F-P) is −15.8 Hz at a distance of 6.22 Å in the complex with ClF and −12.1 Hz in the complex with FH. When the acid is ClH, J(Cl-P) has a value of −3.2 Hz.

**Figure 7:**
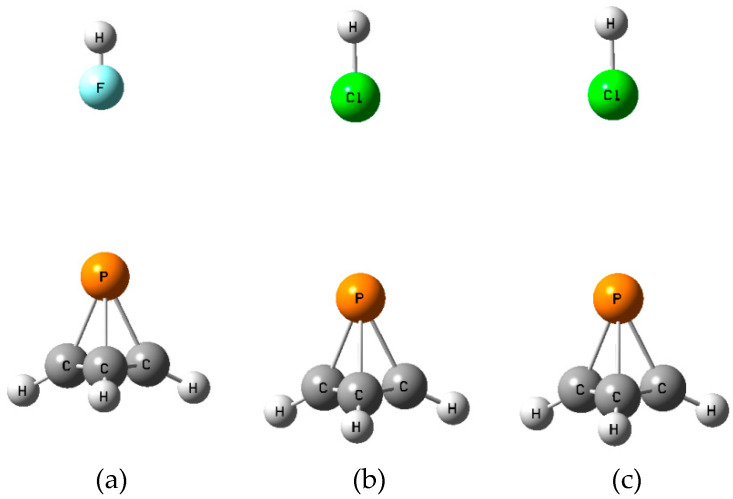
Equilibrium structures **D** for (**a**) FH:P(CH)_3_, (**b**) ClH:P(CH)_3_, and (**c**) FCl:P(CH)_3_.

The structures, total energies, and molecular graphs of the **D** isomers are reported in Table S4 of the Supplementary Materials, and these isomers are illustrated in Figure 7 which shows that the F atom of FH and ClF, and the Cl atom of ClH are adjacent to P. From Figure S3 it can be seen that these complexes have an unfavorable dipole alignment. The binding energies of the **D** isomers are reported in Table 5, and are noticeably less than those of the **C** isomers. The **D** isomers have binding energies that range from 0.8 kJ mol^−1^ for HF:P(CH)_3_ at a F-P distance of 3.49 Å, to 3.9 kJ mol^−1^ for HCl:P(CH)_3_ at a Cl-P distance of 3.63 Å. The binding energy of the FCl:P(CH)_3_ complex is slightly less at 3.6 kJ mol^−1^, but the Cl-P distance of 3.22 Å is much shorter.

The **D** isomers have large spin–spin coupling constants J(X-P), and Table S5 shows that total J is essentially equal to the FC term. The smallest coupling constant with a value of 45 Hz is J(Cl-P) in HCl:P(CH)_3_. HF:P(CH)_3_ has a value of J(F-P) equal to 118 Hz. The largest coupling constant among the **D** isomers is J(F-P) for FCl:P(CH)_3_ with a value of 299 Hz.

The SAPT energies for the **C** and **D** isomers are given in Table 6. The SAPT binding energies are less than the MP2 binding energies. In fact, SAPT predicts that the HF:P(CH)_3_ isomer **D** is unbound with an unfavorable electrostatic interaction energy of 0.5 kJ mol^−1^. As usual, the first-order SAPT exchange term is the destabilizing term. However, the electrostatic term is not the primary stabilizing term in these isomers as it is in the A and B isomers, but rather, it is the second-order dispersion term. In the FH:P(CH)_3_ isomer **C**, the dispersion term cancels the exchange term. In the remaining complexes, it has an absolute value that is no more than 2 kJ mol^−1^ less than the exchange term. All of the other terms are stabilizing, except for the induction term for the **C** and **D** isomers of ClF:P(CH)_3_. The sum of the stabilizing terms leads to binding energies between 3 and 5 kJ mol^−1^ for the **C** isomers, and between 1 and 2 kJ mol^−1^ for the bound **D** isomers.

Up to this point, the discussion of coupling constants has focused on coupling across intermolecular bonds. However, it is also of interest to examine how ^1^J(P-C) for P(CH)_3_ changes, depending on whether the P-C bond interacts or does not interact with FH, ClH, or ClF. The data required for this analysis are given in Table S6. For the **B**, **C**, and **D** isomers, ^1^J(P-C) varies by about ±1 Hz relative to isolated P(CH)_3_. For these, ^1^J(P-C) is essentially independent of bond formation by P(CH)_3_. Bond formation in these isomers occurs at relatively long distances, and does not produce a large enough change in the electron distributions of P and C to produce changes in ^1^J(P-C). This is also evident from the small values of the charge-transfer energies in these isomers.

For the **A** isomers, ^1^J(P-C) coupling constants in complexes with FH and ClH span a slightly greater range compared to the isomers **B**, **C**, and **D**. However, it is the **A** isomer with ClF that exhibits dramatic changes. This is consistent with the stronger halogen bonding interaction between P(CH)_3_ and ClF which leads to a significant lengthening of the P-C bond to 1.946 Å, and changes in the electron density of that bond. The electron density changes are evident from the charge-transfer energies, and from the coupling constant for the interacting P-C bond, which decreases in absolute value from −40 Hz in isolated P(CH)_3_ to −33 Hz in this complex. Although the noninteracting P-C bonds decrease in length by only 0.007 Å, ^1^J(P-C) for these bonds increases in absolute value to −45 Hz in response to the changes in the electron density of this isomer.

### 2.6. Structures A-tr and B-tr

**Figure 8:**
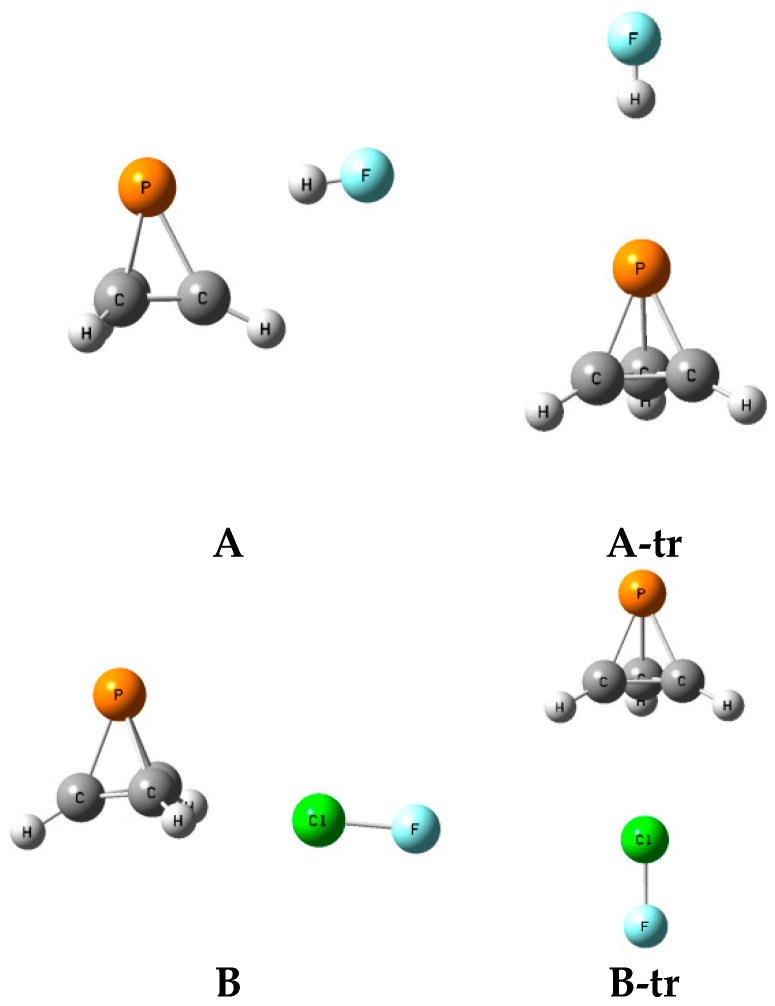
Complexes FH:P(CH)_3_ (**A**) and (**A-tr**), and ClF:P(CH)_3_ (**B**) and (**B-tr**).

The structures, total energies, and molecular graphs of the complexes **A**-tr and **B**-tr are given in Table S7 of the Supplementary Materials, and the structures FH:P(CH)_3_ and FH:P(CH)_3_-**A**-tr, and ClF:P(CH)_3_ and ClF:P(CH)_3_**B**-tr, are illustrated in Figure 8. These complexes have C_3v_ symmetry and are not equilibrium structures on their potential surfaces, since they have two degenerate imaginary frequencies. Rather, complexes labeled **A**-tr are the transition structures for converting one isomer **A** to another one of the **A** isomers, and **B**-tr are the transition structures for converting one isomer **B** to another one of the isomers **B**.

Table 7 presents the binding energies, barriers, and intermolecular distances for the transition structures XY:P(CH)_3_ **A**-tr and **B**-tr. The intermolecular distances in these transition structures are longer than the X-P and X-C distances in the corresponding complexes **A** and **B**, respectively. The **A**-tr structures are bound with stabilization energies ranging from 8 kJ mol^−1^ for FH:P(CH)_3_ **A**-tr and ClH:P(CH)_3_ **A**-tr to 13 kJ mol^−1^ for ClF:P(CH)_3_ **A**-tr. The **B**-tr isomers have binding energies that range from 10 kJ mol^−1^ for FH:P(CH)_3_ **B**-tr to 13 kJ mol^−1^ for ClF:P(CH)_3_ **B**-tr. From these energies of corresponding equilibrium complexes and transition structures, it is possible to evaluate the barrier for converting one **A** or **B** complex to another equivalent complex. These barriers are relatively high for the **A** isomers, and vary from 9 kJ mol^−1^ for ClH:P(CH)_3_ to 19 kJ mol^−1^ for ClF:P(CH)_3_. Given the structure of the **B** isomers, it is not surprising that the barriers to converting one isomer **B** to another **B** are much lower. These barriers vary from 2 kJ mol^−1^ for ClH:P(CH)_3_ to 4 kJ mol^−1^ for FH:P(CH)_3_ and FCl:P(CH)_3_.

## 3. Methods

The structures of the isolated phosphatetrahedrane molecule, P(CH)_3_, the monomers HF, HCl, and ClF, and the complexes formed by phosphatetrahedrane and the monomers were optimized at second-order Møller–Plesset perturbation theory (MP2) [29,30,31,32] with the aug’-cc-pVTZ basis set [33]. This basis set was derived from the Dunning aug-cc-pVTZ basis set [34,35] by removing diffuse functions from H atoms. Frequencies were computed to establish that the optimized structures correspond to equilibrium structures on their potential surfaces. Transition structures that connect two equivalent equilibrium structures were also optimized. Optimization and frequency calculations were performed using the Gaussian 16 program (v 09, Carnegie Mellon University, Pittsburgh, PA, USA) [36]. Complex binding energies (−ΔE) were computed as the negative of the reaction energy for the formation of the complex from phosphatetrahedrane and HF, HCl, or ClF.

The electron density properties at bond critical points (BCPs) of complexes have been analyzed using the Atoms in Molecules (AIM) methodology [37,38,39,40] employing the AIMAll [41] program. The topological analysis of the electron density produces the molecular graph of each complex. This graph identifies the location of electron density features of interest, including the electron density (ρ) maxima associated with the various nuclei, and saddle points which correspond to bond critical points. The zero gradient line which connects a BCP with two nuclei is the bond path. The Natural Bond Order (NBO) [42] method has been used to analyze the stabilizing charge-transfer interactions using the NBO-6 program [43]. In addition, the Electron Localization Function (ELF) [44] has been generated for P(CH)_3_ using the Topmod program [45] to further characterize those regions of space in which the electron density is high.

Density Functional Theory–Symmetry Adapted Perturbation Theory (DFT-SAPT) has been used to investigate interaction energies. In this approach, the energies of interacting monomers are expressed in terms of orbital energies obtained from Kohn–Sham density functional theory [46,47] The SAPT [48] method allows for the decomposition of the interaction energy of a complex into different terms related to physically well-defined components, such as those arising from electrostatic, exchange, induction, and dispersion terms. The interaction energy can be expressed within the framework of the SAPT method as

(1) ΔESAPT = Eel (1)+ Eex(1)+ Eind(2)+ Edisp(2)

where E_el_^(1)^ is the first-order electrostatic interaction energy of the monomers each with its unperturbed electron distribution; E_ex_^(1)^ is the first-order exchange energy term; E_ind_^(2)^ denotes the second-order induction energy arising from the interaction of permanent multipoles with induced multipole moments and charge-transfer contributions, plus the change in the repulsion energy induced by the deformation of the electron clouds of the monomers; E_disp_^(2)^ is the second-order dispersion energy, which is related to the instantaneous multipole-induced multipole moment interactions plus the second-order correction for coupling between the exchange repulsion and the dispersion interactions. In addition to the terms listed in Equation (1), a Hartree–Fock correction term δHF, which includes higher-order induction and exchange corrections, is also included [49]. The DFT-SAPT calculations have been performed with the MOLPRO program [50] at the PBE0/aug’-cc-pVTZ level of theory [51].

Equation of motion coupled cluster singles and doubles (EOM-CCSD) spin–spin coupling constants were evaluated in the CI (configuration interaction)-like approximation [52,53] with all electrons correlated. For these calculations, the Ahlrichs [54] qzp basis set was placed on ^13^C and ^19^F, and the qz2p basis on ^31^P, ^35^Cl, and the ^1^H atom of FH and ClH. The Dunning cc-pVDZ basis set was placed on the ^1^H atoms bonded to C atoms. All terms that contribute to the total coupling constant, namely, the paramagnetic spin orbit (PSO), diamagnetic spin orbit (DSO), Fermi contact (FC), and spin dipole (SD) have been evaluated. Coupling constant calculations were performed using ACES II [55] on the HPC cluster Owens at the Ohio Supercomputer Center.

## 4. Conclusions

Ab initio MP2/aug’-cc-pVTZ calculations have been performed to determine the structures and binding energies of complexes formed by phosphatetrahedrane, P(CH)_3_, and the monomers HF, HCl, and ClF. The following statements are supported by the results of this study.

1. Four types of complexes, designated **A**, **B**, **C**, and **D**, have been found on the potential energy surfaces. Isomers **A** form at the P atom near the end of a P-C bond, **B** at a C-C bond, **C** at the centroid of the C-C-C ring along the C_3_ symmetry axis, and **D** at the P atom along the C_3_ symmetry axis.
2. Complexes **A** and **B** are stabilized by hydrogen bonds when FH and ClH are the acids, and by halogen bonds when ClF is the acid. In isomers **C**, the dipole moments of the two monomers are favorably aligned, and there exists a long-range interaction between the atom at the negative end of the dipole moment vector and the P atom. In the **D** isomers, the dipole moment vectors are unfavorably aligned.
3. The binding energies of the **A** and **B** isomers decrease in the order ClF > FH > ClH, while the binding energies of the **C** and **D** isomers decrease in the order ClH > ClF > HF. For fixed FH, ClH, or ClF, binding energies of the isomers decrease in the order **A** > **B** > **C** > **D**.
4. Charge-transfer stabilizes isomers **A**, but the charge-transfer interactions are small in isomers **B** and **D**, and nonexistent in **C**.
5. The SAPT binding energies for the **A** and **B** isomers are smaller than the corresponding MP2 binding energies. The most stabilizing SAPT component for these isomers is the electrostatic interaction, except for ClH:P(CH)_3_ isomer **B** for which the dispersion interaction is the most stabilizing. These two terms are similar for the **B** isomer of FCl:P(CH)_3_. In contrast, the dispersion interaction is the most stabilizing term for the **C** and **D** isomers.
6. Transition structures for converting one isomer **A** to another **A**, and one isomer **B** to another **B**, have been found on the potential surfaces. The barriers for converting one isomer to another are significantly higher for the **A** isomers.
7. EOM-CCSD coupling constants J(X-C) are small for both the **B** and **C** isomers. J(X-P) values are larger and positive for the **A** isomers, negative for the **B** isomers, and have their largest values for the **D** isomers.
8. Coupling constants ^1^J(P-C) in the isomers change very little from their value in isolated (PH)_3_, except for the complex FCl:P(CH)_3_ isomer **A**. ^1^J(P-C) decreases in absolute value for the P-C bond that interacts with ClF as the P-C bond elongates significantly. ^1^J(P-C) increases in absolute value for the other two P-C bonds, even though there is little change in the P-C distances relative to isolated P(CH)_3_.

## Figures and Tables

**Table 1:** Binding energies (−ΔE, kJ mol^−1^), distances (R, Å), selected angles (<, ^o^), charge-transfer energies (CT, kJ mol^−1^), and spin–spin coupling constants across intermolecular bonds [J(X-P), Hz] for complexes XY:P(CH)_3_ isomers **A**.

Acid	−ΔE	R(X-P) ^a^	R(P-C) ^b^	< 1	< 2	P_lp_→σ*X-Y	J(X-P) ^c^
FH	20.0	3.412	1.881	C-P-F = 65	H-F-P = 5	17.6	28.0
ClH	16.5	3.870	1.875	C-P-Cl = 68	H-Cl-P = 5	16.4	3.3
ClF	31.6	2.552	1.946	C-P-Cl = 77	P-Cl-F = 169	83.1 ^d^, 36.5, 17.0 ^e^	232.0

^a^ R is F-P for the complex with FH and Cl-P for complexes with ClH and ClF. ^b^ The P-C bond that interacts with FH, ClH, and FCl. The P-C bond distance in P(CH)_3_ is 1.857 Å. ^c 2h^J(X-P) for hydrogen-bonded complexes and ^1p^J(Cl-P) for FCl:P(CH)_3_. ^d^ σ(P-C) →σ*Cl-F. ^e^ Cl_lp_→σ*P-C.

**Table 2:** Binding energies (−ΔE, kJ mol^−1^), distances (R(X-γ)^a^ and R(X-C), Å)), angles P-γ-X^a^ (<, ^o^), charge-transfer energies (CT, kJ mol^−1^), and spin–spin coupling constants across intermolecular bonds (J(X-C), Hz) for complexes XY:P(CH)_3_ isomers **B**.

Acid	−ΔE	R(X−C)/R(X−γ) ^a^	<P–γ–X ^a^	σ C–C→σ*X–Y	J(X–C) ^b^
FH	14.8	3.113/2.094	129	1.9	−1.8
ClH	14.1	3.542/2.193	101	1.6	0.1
ClF	17.3	2.920/2.825	121	2.1	0.2

^a^ γ is the midpoint of the interacting C-C bond. ^b 2h^J (X-C) for the hydrogen-bonded complexes and ^1p^J(Cl-C) for FCl:P(CH)_3_.

**Table 3:** SAPT energies (kJ mol^−1^) for equilibrium XY:P(CH)_3_ complexes isomers **A** and **B**.

XY:P(CH)_3_ A XY =	E_el_^(1)^	E_ex_^(1)^	E_ind_^(2)^	E_disp_^(2)^	*δ*HF	−ΔE(SAPT) ^a^
HF	−26.1	34.9	−9.3	−12.2	−6.2	18.8
HCl	−20.9	34.5	−5.3	−15.0	−7.8	14.5
FCl	−134.2	270.1	−56.3	−50.1	−63.9	34.5
XY:P(CH)_3_ B XY =						
HF	−17.8	30.3	−8.4	−11.9	−4.5	12.4
HCl	−11.9	27.4	−4.6	−16.3	−4.2	9.6
ClF	−22.8	48.4	−3.9	−21.4	−9.9	9.5

^a^ −ΔE(SAPT) is the DFT-SAPT interaction energy equal to the negative of the sum of the component energies.

**Table 4:** Binding energies (−ΔE, kJ mol^−1^), intermolecular distances (R, Å), and spin–spin coupling constants (J, Hz) for complexes XY:P(CH)_3_ isomers **C** ^a^.

Acid	−ΔE	R(X-C)	R(X-P)	J(X-C)	J(X-P)
FH	4.5	3.206	4.747	3.2	−12.1
ClH	9.2	3.359	4.905	1.4	−3.2
ClF	6.9	4.645 ^b^	6.220 ^b^	0.0 ^b^	−15.8 ^b^

^a^ There is no charge-transfer in the **C** isomers. ^b^ X is the F atom.

**Table 5:** Binding energies (−ΔE, kJ mol^−1^), intermolecular distances (R, Å), charge-transfer energies (CT, kJ mol^−1^), and spin–spin coupling constants (J, Hz]) for complexes XY:P(CH)_3_ isomers **D**.

Acid	−ΔE	P_lp_→σ*(X-Y)	R(X-P) ^a^	J(X-P) ^a^
FH	0.8	0.6	3.489	118.1
ClH	3.9	2.9	3.627	44.6
ClF	3.6	1.6	3.219	299.3

^a^ X is the atom adjacent to P.

**Table 6:** SAPT energies (kJ mol^−1^) for equilibrium XY:P(CH)_3_ complexes isomers **C** and **D**.

XY:P(CH)_3_ C XY =	E_el_^(1)^	E_ex_^(1)^	E_ind_^(2)^	E_disp_^(2)^	*δ*HF	−ΔE(SAPT) ^a^
HF	−2.1	5.6	−0.4	−5.7	−0.3	3.0
HCl	−5.1	13.2	−0.3	−12.0	−1.1	5.2
FCl	−3.0	9.3	0.0	−8.9	−0.8	3.4
XY:P(CH)_3_ D XY =						
HF	0.5	3.3	−0.3	−2.8	−0.3	−0.2 ^b^
HCl	−2.0	7.1	−0.2	−6.1	−0.7	2.0
ClF	−2.1	7.5	0.0	−5.5	−1.2	1.2

^a^ −ΔE(SAPT) is the DFT-SAPT interaction energy equal to the negative of the sum of the component energies. ^b^ Based on the SAPT analysis, this complex is not bound.

**Table 7:** Binding energies (−ΔE), barriers (ΔE^†^, kJ mol^−1^), and intermolecular distances (R,Å), for the transition structures XY:P(CH)_3_ A-tr and XY:P(CH)_3_ B-tr.

	A-tr			B-tr		
Acid	−ΔE	ΔE ^†^	R(X-P)	−ΔE	ΔE ^†^	R(X-C)
FH	7.8	12.2	3.519	10.4	4.4	3.258
ClH	7.5	9.0	3.965	12.1	2.0	3.653
ClF	12.9	18.7	2.985	13.0	4.3	3.137

## Supplementary Materials

The following are available online, Table S1: Structures (Å), total energies (a.u.), and molecular graphs of P(CH)_3_ and XY:P(CH)_3_ complexes **A**; Table S2: Structures (Å), total energies (a.u.), and molecular graphs of complexes **B**; Table S3: Structures (Å), total energies (a.u.), and molecular graphs of complexes **C**. Table S4: Structures (Å), total energies (a.u.), and molecular graphs of complexes **D**; Table S5: PSO, DSO, FC, and SD components of total J for complexes **A**, **B**, **C**, and **D**; Figure S1: ^2h^J(F-P) (Hz) versus the F-P distance (Å) for FH:P(CH)_3_ **A** along the F-P intrinsic reaction path; Figure S2: ^1x^J(Cl-P) (Hz) versus the Cl-P distance (Å) for FCl:P(CH)_3_ **A** along the Cl-P intrinsic reaction path; Fig: S3. Dipole alignments in the isomers **C** and **D** for ClH:P(CH)_3_;Table S6: ^1^J(P-C) (Hz) for P-C bonds of P(CH)_3_ that do or do not interact with FH, ClH, and ClF in complexes **A**, **B**, **C**, and **D**; Table S7: Structures (Å), total energies (a.u.), and molecular graphs of transition structures.
