# Supplementary material for: Unusual Complexes of P(CH)3 with FH, ClH, and ClF
Source: Molecules. 2020 Jun 19;25(12):2846. doi: 10.3390/molecules25122846 (PMC7356268; doi:10.3390/molecules25122846)
Supplement: Supplementary file 1 [file molecules-25-02846-s001.pdf]

Supporting Information for

Unusual Complexes of $P(CH)_3$ with FH, ClH, and FCl

Janet E. Del Bene,[a] Ibon Alkorta,[b] and José Elguero[b]

[a] *Department of Chemistry, Youngstown State University, Youngstown, Ohio 44555, USA*

[b] *Instituto de Química Médica (IQM-CSIC), Juan de la Cierva, 3, E-28006 Madrid, Spain*

Pgs. S2 Table S1. Structures (Å), total energies (a.u.), and molecular graphs of $P(CH)_3$ and $XY{:}P(CH)_3$ complexes **A**.

Pg. S3 Table S2. Structures (Å), total energies (a.u.), and molecular graphs of complexes **B**.

Pg. S4 Table S3. Structures (Å), total energies (a.u.), and molecular graphs of complexes **C**.

Pg. S5 Table S4. Structures (Å), total energies (a.u.), and molecular graphs of complexes **D**.

Pg. S6 Table S5. PSO, DSO, FC, and SD components of total J for complexes **A**, **B**, **C**, and **D**.

Pg. S7 Fig. S1 ^{2h}J(F-P) (Hz) versus the F-P distance (Å) for $FH{:}P(CH)_3$ **A** along the F-P intrinsic reaction path.

Pg. S7 Fig. S2. ^{1x}J(Cl-P) (Hz) versus the Cl-P distance (Å) for $FCl{:}P(CH)_3$ **A** along the Cl-P intrinsic reaction path.

Pg. S8 Fig. S3. Dipole alignments in the isomers **C** and **D** for $ClH{:}P(CH)_3$.

Pg. S9 Table S6. ^{1}J(P-C) (Hz) for P-C bonds of $P(CH)_3$ that do or do not interact with FH, ClH, and ClF in complexes **A**, **B**, **C**, and **D**.

Pgs. S10 – 11 Table S7. Structures (Å), total energies (a.u.), and molecular graphs of transition structures.

Table S1. Structures (Å), total energies (a.u.), and molecular graphs of $P(CH)_3$ and $XY{:}P(CH)_3$ complexes **A**.

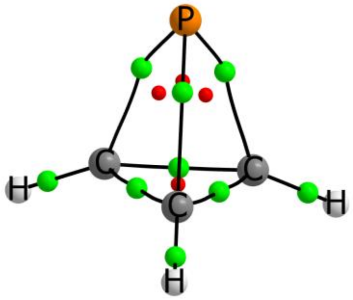	C_3H_3P<br>MP2= -456.63885880 NIMAG= 0<br>P,0.,0.,1.4295144635<br>C,0.8458105226,0.,-0.223504098<br>C,-0.4229052614,-0.7324933993,-0.223504098<br>C,-0.4229052613,0.7324933994,-0.223504098<br>H,1.8094069194,-0.0000000001,-0.6918739831<br>H,-0.9047034598,-1.5669923579,-0.6918739831<br>H,-0.9047034596,1.566992358,-0.6918739831
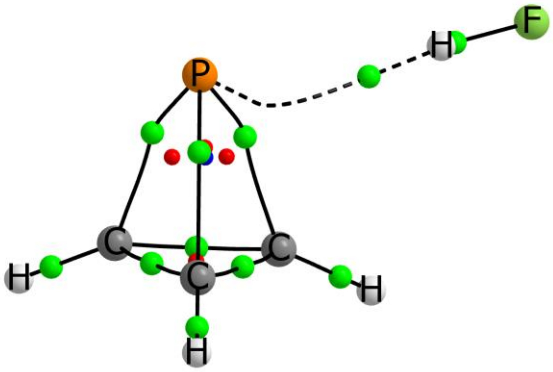	$C_3H_3P{:}HF$ (A)<br>MP2= -556.98688314 NIMAG= 0<br>P,-1.0055179229,-1.0177023062,0.<br>C,0.8751029552,-1.0343136986,0.<br>C,0.2718088206,-2.1423868938,0.7341450432<br>C,0.2718088206,-2.1423868938,-0.7341450432<br>H,1.741208448,-0.4006724264,0.<br>H,0.4528506334,-2.7845227668,1.5729967012<br>H,0.4528506334,-2.7845227668,-1.5729967012<br>H,0.1285827285,1.1932291285,0.<br>F,0.4417948599,2.0722790074,0.
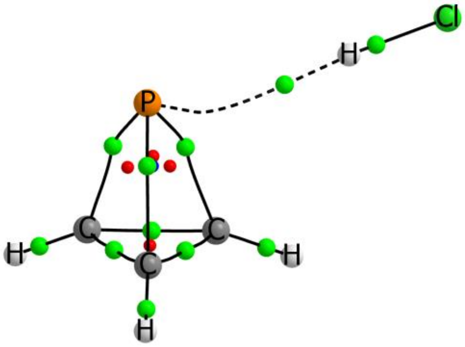	$C_3H_3P{:}HCl$ (A)<br>MP2= -916.95959525 NIMAG= 0<br>P,-1.0143747249,-0.9638798373,0.<br>C,0.8603298618,-0.9602114368,0.<br>C,0.2736821604,-2.0785289105,0.7340623326<br>C,0.2736821604,-2.0785289105,-0.7340623326<br>H,1.715668339,-0.3126356017,0.<br>H,0.4651602132,-2.7202865757,1.5709087139<br>H,0.4651602132,-2.7202865757,-1.5709087139<br>H,0.0366255889,1.4015621816,0.<br>Cl,0.4123611962,2.6337194994,0.
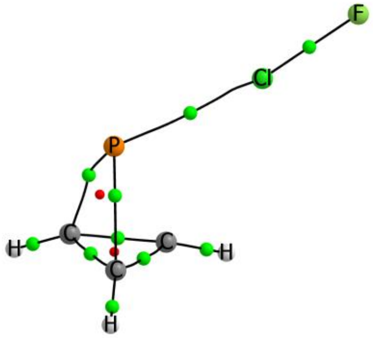	$C_3H_3P{:}ClF$ (A)<br>MP2= -1016.01271690 NIMAG= 0<br>P,-0.9112111459,-0.926383996,0.<br>C,1.0211821078,-1.1528021619,0.<br>C,0.2753671016,-2.131112393,0.7492444775<br>C,0.2753671016,-2.131112393,-0.7492444775<br>H,1.8884276354,-0.5225049089,0.<br>H,0.385036045,-2.8127755103,1.5706941822<br>H,0.385036045,-2.8127755103,-1.5706941822<br>Cl,-0.0528338906,1.4770459264,0.<br>F,0.2219240067,3.2133447917,0.

Table S2. Structures (Å), total energies (a.u.), and molecular graphs of complexes **B**.

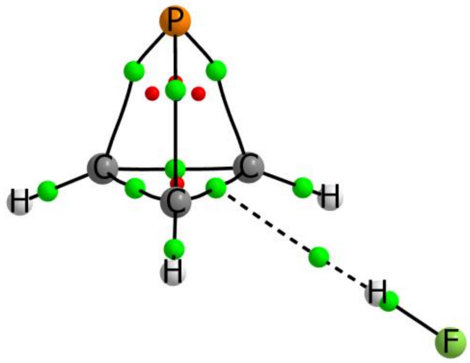	C_3H_3P:HF (B)<br>MP2= -556.98488324 NIMAG= 0<br>P,-0.3234405614,-0.560215486,1.5724251219<br>C,0.5197977619,-0.5780589497,-0.0797718927<br>C,-0.7519199942,-1.3023636265,-0.0703303818<br>C,-0.7605126141,0.1611285952,-0.0797718916<br>H,1.4852916728,-0.6173435155,-0.5472343005<br>H,-1.2325538058,-2.1348458022,-0.5440356889<br>H,-1.2772809977,0.9776285694,-0.5472342982<br>H,0.826961738,1.432339737,-0.9717318675<br>F,1.2462730908,2.1586083003,-1.3742375395
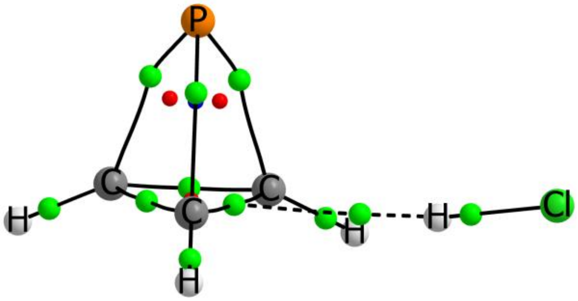	C_3H_3P:HCl (B)<br>MP2= -916.95870314 NIMAG= 0<br>P,1.279343667,0.1515359203,0.<br>C,-0.4123867687,0.3674890598,0.7373131667<br>C,0.0534117854,1.5426714084,0.<br>C,-0.4123867687,0.3674890598,-0.7373131667<br>H,-1.0213695062,0.1083327806,1.5812965696<br>H,-0.0436471277,2.6100898719,0.<br>H,-1.0213695062,0.1083327806,-1.5812965696<br>H,-1.0917119063,-1.7171933778,0.<br>Cl,-1.2920648715,-2.9832004927,0.
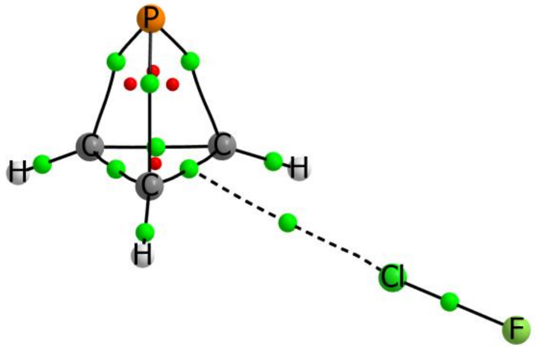	C_3H_3P:ClF (B)<br>MP2= -1016.00727079 NIMAG= 0<br>P,1.5123150225,0.6284420037,0.<br>C,-0.1815604127,0.4740829491,0.7392147198<br>C,0.0126920872,1.7222583333,0.<br>C,-0.1815604127,0.4740829491,-0.7392147198<br>H,-0.7085498328,0.1005203361,1.5947715348<br>H,-0.3109127254,2.7441192099,0.<br>H,-0.7085498328,0.1005203361,-1.5947715348<br>Cl,-1.4128802635,-2.0688600487,0.<br>F,-1.9831746348,-3.619619069,0.

Table S3. Structures (Å), total energies (a.u.), and molecular graphs of complexes **C**.

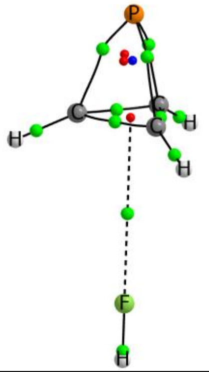	C_3H_3P:HF (C)<br>P,0.,0.0000000015,-2.2930997077<br>C,0.0000000007,0.8450219416,-0.6389295636<br>C,-0.7318104672,-0.4225109679,-0.6389295636<br>C,0.7318104665,-0.4225109691,-0.6389295636<br>H,0.0000000014,1.804188809,-0.1617407106<br>H,-1.5624733412,-0.902094401,-0.1617407106<br>H,1.5624733398,-0.9020944034,-0.1617407106<br>H,0.,0.0000000015,3.3763803373<br>F,0.,0.0000000015,2.4536913445
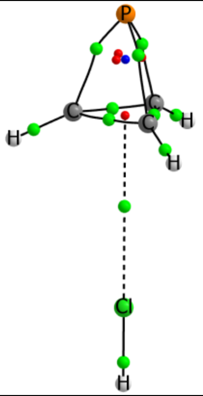	C_3H_3P:HCl (C)<br>MP2= -916.95681811 NIMAG= 0<br>P,0.,0.0000000013,-2.32755043<br>C,0.0000000008,0.84551977,-0.67376968<br>C,-0.7322415995,-0.4227598823,-0.67376968<br>C,0.7322415987,-0.4227598838,-0.67376968<br>H,0.0000000018,1.80920695,-0.2047063<br>H,-1.5668191792,-0.9046034715,-0.2047063<br>H,1.5668191774,-0.9046034746,-0.2047063<br>H,0.,0.0000000013,3.85332751<br>Cl,0.,0.0000000013,2.57714913
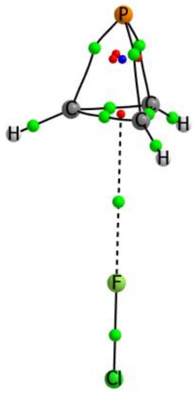	C_3H_3P:ClF (C)<br>MP2= -1016.00333295 NIMAG= 0<br>P,0.,0.,2.0302324399<br>C,0.000000112,0.8456976665,0.377053738<br>C,0.7323956072,-0.4228489302,0.377053738<br>C,-0.7323957191,-0.4228487363,0.377053738<br>H,0.0000002394,1.8084451218,-0.0934697089<br>H,1.5661592972,-0.9042227683,-0.0934697089<br>H,-1.5661595366,-0.9042223536,-0.0934697089<br>F,0.,0.,-2.5486922823<br>Cl,0.,0.,-4.1898670906

Table S4. Structures (Å), total energies (a.u.), and molecular graphs of complexes **D**.

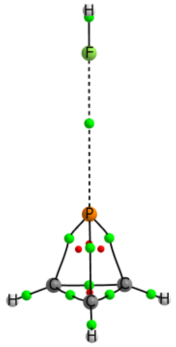	C_3H_3P:HF (D)<br>MP2= -556.97957100 NIMAG= 0<br>P,0.,0.,1.1020677007<br>C,0.8455205236,0.,-0.553230496<br>C,-0.4227602618,-0.7322422528,-0.553230496<br>C,-0.4227602617,0.7322422528,-0.553230496<br>H,1.8095183173,-0.0000000001,-1.0210647542<br>H,-0.9047591587,-1.5670888313,-1.0210647542<br>H,-0.9047591586,1.5670888314,-1.0210647542<br>F,0.,0.,4.591035106<br>H,0.,0.,5.5131672372
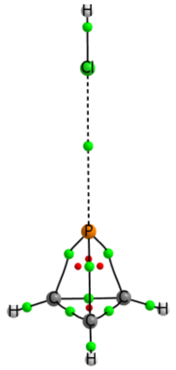	C_3H_3P:HCl (D)<br>MP2= -916.95480177 NIMAG= 0<br>P,0.,0.0000000968,-0.2259019695<br>C,0.0000000879,0.845721393,-1.8791303615<br>C,0.7324160831,-0.4228606274,-1.8791303615<br>C,-0.732416171,-0.4228604752,-1.8791303615<br>H,0.0000001882,1.8105925738,-2.3453121734<br>H,1.5680189869,-0.9052963047,-2.3453121734<br>H,-1.5680191751,-0.9052959787,-2.3453121734<br>Cl,0.,0.0000000968,3.4008399116<br>H,0.,0.0000000968,4.6765093277
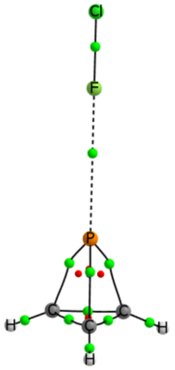	C_3H_3P:ClF (D)<br>MP2= -1016.00205000 NIMAG= 0<br>P,0.,0.0000000968,-0.1751656791<br>C,0.0000000879,0.8455660291,-1.8296854362<br>C,0.732281534,-0.4227829455,-1.8296854362<br>C,-0.732281622,-0.4227827932,-1.8296854362<br>H,0.0000001883,1.8108733627,-2.2949353228<br>H,1.5682621572,-0.9054366993,-2.2949353228<br>H,-1.5682623455,-0.9054363731,-2.2949353228<br>F,0.,0.0000000968,3.0433714043<br>Cl,0.,0.0000000968,4.6837933739

Table S5. PSO, DSO, FC, and SD components of total J for complexes **A**, **B**, **C**, and **D**.

Complexes of P(CH)3 with

	PSO	DSO	FC	SD	J
HF **A**	-4.2	0.0	31.3	1.8	28.9[a]
HCl **A**	-0.5	0.0	3.5	0.3	3.3[a]
ClF **A**	6.7	0.0	222.4	2.8	232.0[b]
HF **B**	-2.4	-0.2	0.1	0.6	-1.8[c]
HCl **B**	0.0	0.0	0.0	0.1	0.1[c]
ClF **B**	0.6	0.0	-0.5	0.1	0.2[d]
HF **C**	-0.2	-0.3	-11.7	0.1	-12.1[e]
HCl **C**	0.2	0.0	-3.5	0.2	-3.2[f]
ClF **C**	4.8	-0.2	-21.5	1.1	-15.8[e]
HF **C**	-0.1	0.2	3.1	-0.1	3.2[g]
HCl **C**	0	0	1.4	0	1.4[h]
ClF **C**	0.2	0.4	5.0	-0.2	5.4[g]
HF **D**	-0.6	0.3	118.4	0.1	118.1[e]
HCl **D**	0.0	0.0	44.6	0.0	44.6[f]
ClF **D**	0.4	0.5	298.6	-0.2	299.3[e]
HF **A**-tr	-1.3	0.1	69.3	0.6	68.7[a]
HCl **A**-tr	-0.1	0.0	7.0	0.1	7.0[a]
ClF **A**-tr	0.6	0.0	257.0	-0.5	257.1[b]
HF **B**-tr	-10.4	-0.3	-8.9	0.5	-19.2[a]
HCl **B**-tr	-1.7	0.0	-1.1	0.1	-2.7[a]
ClF **B**-tr	5.2	0.0	-7.7	1.5	-1.0[b]

a) ${}^{2h}J$(X-P); b) ${}^{1x}J$(Cl-P); c) ${}^{2h}J$(X-C); d) ${}^{1x}J$(Cl-C); e) J(F-P); f) J(Cl-P); g) J(F-C); h) J(Cl-C); i) J(F-P); J(Cl-P).

Fig. S1 $^{2h}J(F\text{-}P)$ (Hz) versus the F-P distance (Å) for FH:P(CH)$_3$ **A** along the F-P intrinsic reaction path.

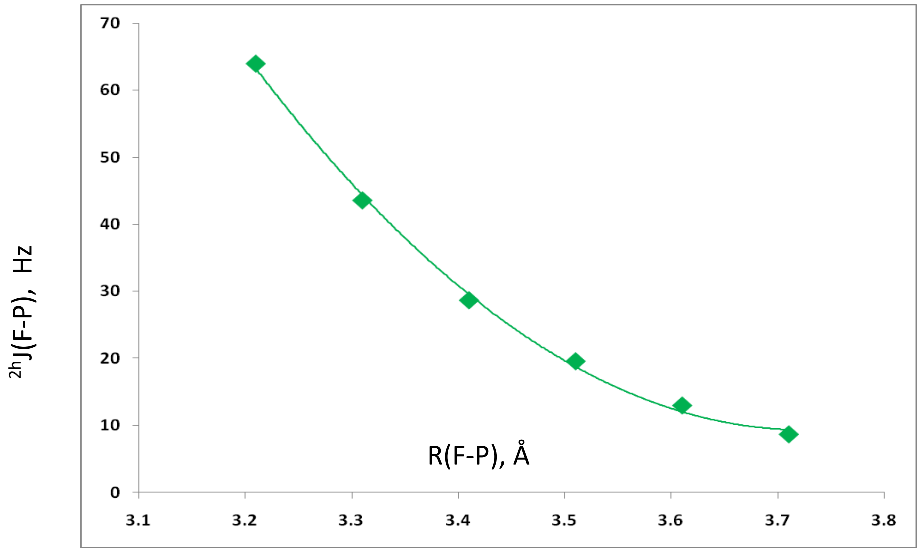

Fig. S2. $^{1x}J(Cl\text{-}P)$ (Hz) versus the Cl-P distance (Å) for FCl:P(CH)$_3$ **A** along the Cl-P intrinsic reaction path.

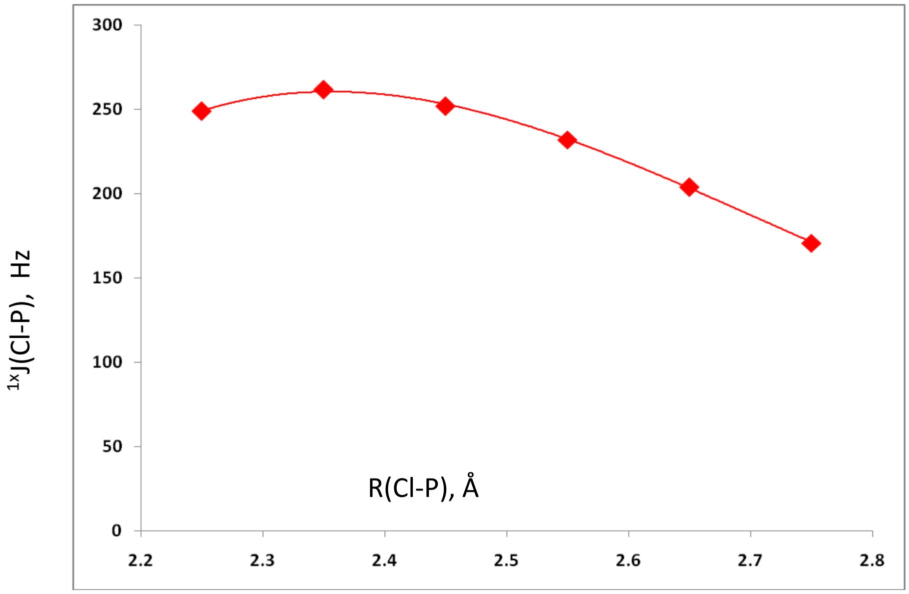

Fig. S3. Dipole alignments in the isomers **C** and **D** for $ClH{:}P(CH)_3$.

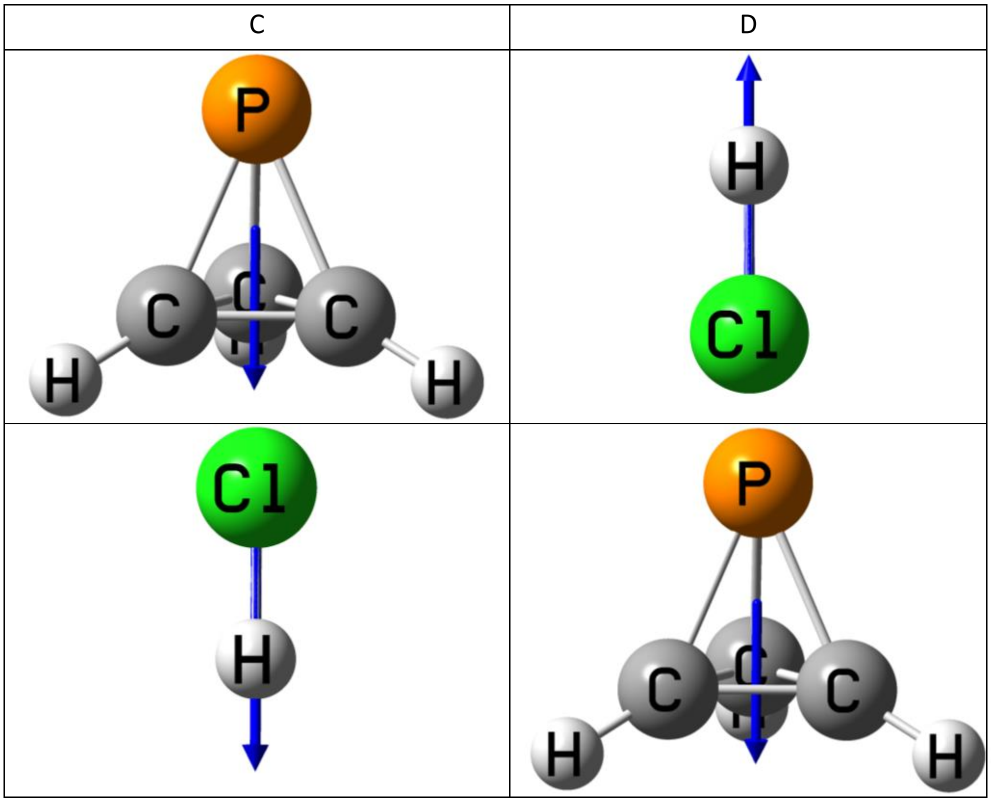

Table S6. ^1J(P-C) (Hz) for P-C bonds of $P(CH)_3$[a] that do or do not interact with FH, ClH, and ClF in complexes **A**, **B**, **C**, and **D**.

**A**	^1J(P-C) interacting	^1J(P-C) noninteracting
FH	-42.5	-39.1
ClH	-40.5	-39.6
ClF	-33.1	-45.2

**B**	^1J(P-C) interacting	^1J(P-C) noninteracting
FH	-40.0	-41.4
ClH	-39.6	-41.5
ClF	-39.9	-40.7

**C**	^1J(P-C) interacting	
FH	-39.3	
ClH	-40.1	
ClF	-39.6	

**D**		^1J(P-C) noninteracting
FH		-41.5
ClH		-40.1
ClF		-40.9

a) ^1J(P-C) = -40.3 Hz for $P(CH)_3$.

Table S7. Structures (Å), total energies (a.u.), and molecular graphs of transition structures.

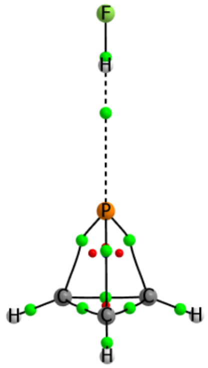	C_3H_3P:HF (A-tr)<br>MP2= -556.98222723 NIMAG= 2<br>P,0.,0.,1.2919511674<br>C,0.8467120193,0.,-0.3521192519<br>C,-0.4233560097,-0.7332741184,-0.3521192519<br>C,-0.4233560096,0.7332741184,-0.3521192519<br>H,1.8125954318,-0.0000000001,-0.8164477897<br>H,-0.9062977159,-1.5697536907,-0.8164477897<br>H,-0.9062977158,1.5697536907,-0.8164477897<br>H,0.,0.,3.8859568023<br>F,0.,0.,4.8111772953
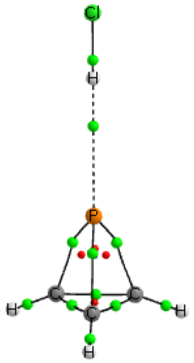	C_3H_3P:HCl (A-tr)<br>MP2= -916.95616998 NIMAG= 2<br>P,0.,0.0000000968,-0.0217254821<br>C,0.000000088,0.8462672657,-1.669655022<br>C,0.7328888227,-0.4231335639,-1.669655022<br>C,-0.7328889107,-0.4231334115,-1.669655022<br>H,0.0000001885,1.8124983648,-2.1332601138<br>H,1.5696694501,-0.9062492004,-2.1332601138<br>H,-1.5696696386,-0.906248874,-2.1332601138<br>H,0.,0.0000000968,2.6650485632<br>Cl,0.,0.0000000968,3.9435600388
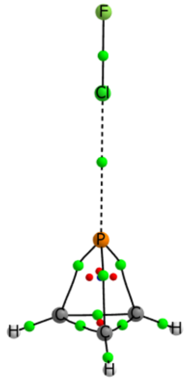	C_3H_3P:ClF (A-tr)<br>MP2= -1016.00558891 NIMAG= 2<br>P,0.,0.0000000968,-0.1313005907<br>C,0.000000088,0.8471471664,-1.7754121881<br>C,0.733650839,-0.4235735143,-1.7754121881<br>C,-0.7336509271,-0.4235733618,-1.7754121881<br>H,0.0000001884,1.812259673,-2.2414009503<br>H,1.5694627371,-0.9061298545,-2.2414009503<br>H,-1.5694629255,-0.9061295282,-2.2414009503<br>Cl,0.,0.0000000968,2.8539126848<br>F,0.,0.0000000968,4.5059643204
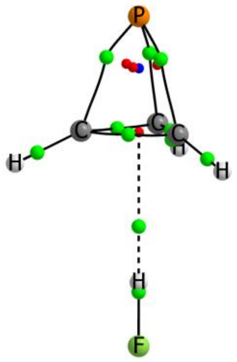	C3H3P:HF (B-tr)<br>MP2= -556.98320288 NIMAG= 2<br>P,0.,0.0000000015,-2.0895876044<br>C,0.0000000007,0.8491215134,-0.4434190667<br>C,-0.7353608005,-0.4245607538,-0.4434190667<br>C,0.7353607999,-0.424560755,-0.4434190667<br>H,0.0000000014,1.8206034388,0.0112489076<br>H,-1.5766888276,-0.9103017159,0.0112489076<br>H,1.5766888262,-0.9103017184,0.0112489076<br>F,0.,0.0000000015,2.7022006281<br>H,0.,0.0000000015,1.7762427603

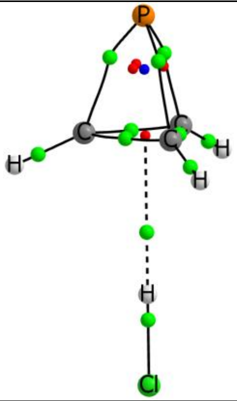	C3H3P:HCl (B-tr)<br>MP2= -916.95794111 NIMAG= 2<br>P,0.,0.,1.8394820606<br>C,0.0000001123,0.8480132664,0.1900112472<br>C,0.7344009754,-0.4240067305,0.1900112472<br>C,-0.7344010876,-0.424006536,0.1900112472<br>H,0.0000002409,1.819408126,-0.2643950305<br>H,1.5756535365,-0.9097042716,-0.2643950305<br>H,-1.5756537774,-0.9097038544,-0.2643950305<br>H,0.,0.,-2.0841074453<br>Cl,0.,0.,-3.3632057642
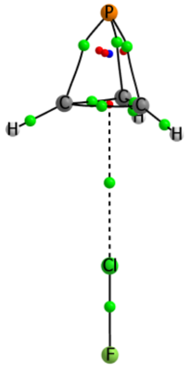	C3H3P:ClF (B-tr)<br>MP2= -1016.00564083 NIMAG= 2<br>P,0.,0.,2.0438283848<br>C,0.0000001122,0.8477370391,0.3924675819<br>C,0.7341617555,-0.4238686168,0.3924675819<br>C,-0.7341618677,-0.4238684224,0.3924675819<br>H,0.0000002407,1.8181002777,-0.0634980417<br>H,1.5745209068,-0.9090503473,-0.0634980417<br>H,-1.5745211475,-0.9090499304,-0.0634980417<br>Cl,0.,0.,-2.6278408517<br>F,0.,0.,-4.2709801826
